# Gait Assessment of Pain and Analgesics: Comparison of the DigiGait™ and CatWalk™ Gait Imaging Systems

**DOI:** 10.1007/s12264-018-00331-y

**Published:** 2019-01-18

**Authors:** Yu Xu, Na-Xi Tian, Qing-Yang Bai, Qi Chen, Xiao-Hong Sun, Yun Wang

**Affiliations:** 1 Neuroscience Research Institute and Department of Neurobiology, School of Basic Medical Sciences, Key Laboratory for Neuroscience, Ministry of Education/National Health Commission, Peking University, Beijing, 100083 China; 2 PKU-IDG/McGovern Institute for Brain Research, Peking University, Beijing, 100871 China; 3 Department of Neurobiology, Capital Medical University, Beijing, 100069 China

**Keywords:** Gait analysis, DigiGait™, CatWalk™, Neuropathic pain, Inflammatory pain, Analgesic

## Abstract

Investigation of pain requires measurements of nociceptive sensitivity and other pain-related behaviors. Recent studies have indicated the superiority of gait analysis over traditional evaluations (e.g., skin sensitivity and sciatic function index [SFI]) in detecting subtle improvements and deteriorations in animal models. Here, pain-related gait parameters, whose criteria include (1) alteration in pain models, (2) correlation with nociceptive threshold, and (3) normalization by analgesics, were identified in representative models of neuropathic pain (spared nerve injury: coordination data) and inflammatory pain (intraplantar complete Freund’s adjuvant: both coordination and intensity data) in the DigiGait™ and CatWalk™ systems. DigiGait™ had advantages in fixed speed (controlled by treadmill) and dynamic SFI, while CatWalk™ excelled in intrinsic velocity, intensity data, and high-quality 3D images. Insights into the applicability of each system may provide guidance for selecting the appropriate gait imaging system for different animal models and optimization for future pain research.

**Electronic supplementary material:**

The online version of this article (10.1007/s12264-018-00331-y) contains supplementary material, which is available to authorized users.

## Introduction

In human society, the great burden of chronic pain mainly derives from extensive disability [1]. In particular, low back pain topped the leading causes of worldwide disability in the 2015 Global Burden of Disease Study [2]. Physical inactivity may be a reflection or consequence of persistent pain and mental distress, and the trigger or contributor of pain-induced disability [3], indicating the importance of motor evaluation in both clinical and basic pain research.

Among numerous behavioral paradigms in pain models [4, 5], escape reactions evoked by mechanical or thermal stimuli are generally acknowledged and applied [6–10], yet their non-standardized testing protocols and definitions of a withdrawal reaction limit their reliability and validity. Compared to sensory tests, experiments evaluating higher-order functions are time- and labor-intensive and require larger sample sizes on account of variable responses. In addition, many experiments involving emotion (e.g., open field, elevated plus maze, and forced swimming) cannot be performed repetitively due to the influence of learning and memory from previous trials, limiting their application in self-controlled studies [4]. Thus, it is desirable to establish a relatively simple and accessible system to objectively and repetitively evaluate pain-induced functional abnormalities in animal models.

Active (spontaneous) and passive (forced) gait analyses have been performed using various laboratory models of peripheral inflammatory, neuropathic, and cancer pain [11–13]. Our previous study confirmed the efficiency of the CatWalk^™^ gait system in revealing subtle gait alterations after intrathecal injection of an analgesic transmembrane peptide [14]. Furthermore, gait acquisition systems may be superior to traditional assessments of skin sensitivity and the sciatic function index (SFI) in (1) detecting abnormalities in advance [15, 16], (2) reflecting subtle deterioration due to increased injury severity [17], and (3) exposing fine improvements due to analgesics [18, 19].

Three platforms, CatWalk^™^ (Noldus Inc., Wageningen, The Netherlands), DigiGait^™^ (Mouse Specifics Inc., Framingham, MA), and TreadScan^™^ (CleverSys Inc., Reston, VA), have been developed and are widely used to acquire gait information from rodents. Of these, DigiGait^™^ and TreadScan^™^ have similar equipment, including a treadmill driving rodents into passive walking/running [20], while a stable track in CatWalk^™^ enables rodents to actively move forward and simultaneously transform pressure into green fluorescence (Illuminated Footprints^™^), and thereby the intensity data of footprints are acquired *via* calculating their brightness [21].

To evaluate pain-induced motor impairment, it is of great utility to be able to select an appropriate gait system from these two categories: (1) treadmill and (2) pressure-fluorescence transforming catwalk track. Herein, in representative models of chronic neuropathic pain (spared nerve injury; SNI) and inflammatory pain (intraplantar complete Freund’s adjuvant; CFA), we compared the efficacy of several major parameters of DigiGait^™^ and CatWalk^™^ in reflecting pain severity, and their sensitivity to treatment with Food and Drug Administration (FDA)-approved analgesics (pregabalin [PGB] and tramadol) in a placebo-controlled, self-controlled, and counterbalanced experimental paradigm [22]. The principal objectives of this study included comparisons between DigiGait^™^ and CatWalk^™^ in (1) illustrating and measuring gait in a comprehensive, quantitative, and objective manner, (2) revealing motor dysfunction and its correlation with pain severity in SNI and CFA models, and (3) detecting subtle improvement of gait parameters in response to analgesic treatment.

## Materials and Methods

### Animals

Male Sprague-Dawley rats at the specific pathogen-free (SPF) level, weighing 150 g were used. All experimental protocols acquired approval from the Animal Care and Use Committee of Peking University. The animals were housed in individual ventilated cages (3–5 rats/cage) on a 12/12-h light-dark cycle with free access to food and water in an SPF laboratory.

### Drugs

The analgesics used in this study were 25 mg/kg PGB (MedChemExpress, Monmouth Junction, NJ) in SNI rats and 30 mg/kg tramadol (Millipore Sigma, Darmstadt, Germany) in CFA rats. The drugs were diluted in normal saline.

### Experimental Design

The behavioral experiments were conducted in a placebo-controlled, self-controlled, and counterbalanced manner to minimize the confounding influence of the placebo effect of analgesics, individual differences, and sequential order, respectively.

Usually, SNI rats show a substantial decline of mechanical threshold in the first week after injury, and this hypersensitivity continues for months [23]. Thus, we performed sensory and motor analyses at 7 days and 9 days post-injury (dpi), when the models showed stable mechanical allodynia. In total, 24 rats received the SNI operation, two of which failed to satisfy the inclusion criteria of marked weight loss and severe immobility. The remaining 22 rats were randomly divided into 2 groups at 7 dpi. All 22 rats, at both 7 and 9 dpi, were injected intraperitoneally (i.p.) with saline (placebo control) or PGB (an FDA-approved analgesic for neuropathic pain). The time between saline and drug injections was ~4 h. In a time-window ranging from 1 h to 2 h after each injection, two types of sensory test (punctate and dynamic allodynia) together with one type of gait analysis (DigiGait^™^ or CatWalk^™^) were performed. Group 1 received DigiGait^™^ at 7 dpi and CatWalk^™^ at 9 dpi, while motor assessments on group 2 were conducted in a counterbalanced fashion.

Intraplantar injection of CFA induces instant thermal and mechanical hyperalgesia within 1 h; this hypersensitivity lasts for two weeks and then gradually subsides [24–26]. Thus, we performed sensory and motor analyses at 7 and 9 dpi, when the models showed stable mechanical allodynia. An intraplantar CFA injection was given to 24 rats, 2 of which met the exclusion criterion of undetectable allodynia. Next, the remaining 22 rats were randomly divided into 2 groups at 7 dpi and the subsequent procedures were identical to the SNI experiments, except for the analgesic, which was replaced by tramadol due to its favorable analgesic action compared to PGB. The experimental paradigm for the CFA model is shown Fig. 5A.

To achieve the goal of completing sensory and gait measurements within 1 h, saline or analgesic was injected in sequence at 15-min intervals. In this way, we at most had to handle 4 rats simultaneously. In addition, to save time, we pre-trained these rats to perform uninterrupted runs on the CatWalk track and DigiGait treadmills and started the environmental habituation for sensory tests on the metal mesh floor 10 min before the 1 h–2 h time window. Most of the rats completed these tests within 1 h, and the few uncooperative individuals were excluded.

### Spared Nerve Injury Model

Under long-term anesthesia induced by 1% pentobarbital sodium (i.p.), the skin on the lateral side of left thigh was shaved and disinfected with 75% ethanol. A 2-cm skin incision was made to expose the left sciatic nerve, and the three branches were separated using a glass dissecting needle. Of these, the tibial and common peroneal nerves were ligated and cut, followed by removal of 5 mm segments to avoid regeneration. At 7 dpi, animals with marked weight loss or severe immobility were excluded.

### CFA-Induced Inflammatory Pain Model

Under short-term anesthesia induced by inhalation of isoflurane (RWD, Shenzhen, China), after disinfection of the skin of the left hindpaw with 75% ethanol, 0.1 mL CFA (Millipore Sigma, Darmstadt, Germany) was injected subcutaneously in the center of the hindpaw followed by gentle pressure to avoid leakage. Six hours after injection, animals with slight or undetectable mechanical allodynia were excluded.

### Punctate Allodynia (von Frey) Measurement

The paw-withdrawal threshold in response to a mechanical stimulus was determined using a series of von Frey fibers (Stoelting, Wood Dale, IL), ranging from 1.00 g to 15.00 g. Animals were placed on a metal mesh floor under a plastic cage and allowed to move freely. Accustomed to this environment for ~10 min prior to testing, rats received von Frey tests on the lateral (SNI) or mid-plantar (CFA) surface of the affected left hindpaw through the mesh floor. Probing was only performed when the animal’s paw was in contact with the floor. Each probe was applied to the foot until it just bent, and was kept in this position for 6 s–8 s [27]. The interval between consecutive filaments was at least 3 min. The first stimulus was 2 g, and the following stimuli were decided according to the up-and-down method [27]. The mechanical threshold was calculated by the formula used in previous studies [27].

### Dynamic Allodynia (Brush) Measurement

Dynamic allodynia was evaluated by light stroking from heel to toe with a paintbrush (velocity ~2 cm/s). The habituation method and the stimulus location were identical to the von Frey measurement described above. Naive rats usually did not respond (score 0). After nerve injury or inflammation, several pain-suggestive responses were observed: rapid lifting of the stimulated paw for < 1 s (score 1); sustained lifting (> 2 s) of the stimulated paw or lifting followed by a horizontal movement of the paw to avoid the aversive stroking (score 2); or one strong lateral paw lift above the level of the body (a lateral kick, more resembling an exaggerated hindpaw withdrawal than a flinch), a startle-like jump, multiple flinching responses, or licking of the affected paw (score 3). This test was repeated three times at intervals of at least 3 min and the average score was calculated for each rat. The paintbrush was prepared by trimming the tip and making it blunt. The total length of the brush was ~5 mm [28].

### CatWalk^™^ XT Analysis

The CatWalk^™^ XT gait system (abbreviated here as CatWalk^™^) consists of an enclosed walkway on a glass plate allowing a rodent to move from one side to the other [19]. Green light enters at the long edge of the plate and is completely internally reflected. Light is able to escape only at areas where the paws contact the glass plate, and the brightness of the scattered light is correlated with the contact intensity (Illuminated Footprint^™^, Noldus). The CatWalk system includes a high-speed digital camera underneath the walkway with a sample rate of 100 frames per second. The camera lens has a diameter of 8.5 mm and a curvature of 65°. The brightness of a pixel depends on the amount of light received from an area by the camera. Illuminated Footprint™ enables the intensity difference between an animal’s paws to be detected.

The 3D footprint intensity tab plots the print intensity of the 4 paws in each individual frame in which the paws contact the glass plate in a 3D chart. The intensity varies from 0 to 255 and is represented by different colors. The 3D chart can be rotated in all directions.

Before experiments, animals were trained to make uninterrupted runs within 3.0 s. Measurements were taken at 7 days or 9 days after SNI surgery or CFA injection. Each rat was placed on the walkway repeatedly at intervals of at least 5 min to complete three uninterrupted runs for further analyses.

Analyses of these recordings yielded many parameters, of which the following have been the most acknowledged and applied in pain models [19]: (1) Coordination data (CatWalk-swing and CatWalk-duty cycle). CatWalk-swing is the duration of the swing phase during walking. Since the durations of the stance and swing phases depend on the walking speed and degree of dysfunction, these parameters are transformed to a fraction of the total step duration according to the following formula: CatWalk-duty cycle = (time in stance phase/time in single step). (2) Area data (CatWalk-max contact area and CatWalk-print area). CatWalk-max contact area is the maximum of total floor area in cm^2^ from single images of the paw in the stance phase. CatWalk-print area is the total floor area in cm^2^ summed from all images of the paw in the stance phase (compared to max contact area, this parameter reflects the entire paw area from the third toe to the heel). (3) Intensity data (CatWalk-mean intensity). CatWalk-mean intensity is the mean intensity of the contact area of the hindpaw during a step cycle. This parameter is expressed in arbitrary units.

To clearly illustrate the alteration of the intensity and print area data for the inflamed left hindpaw, we used the formula left hindpaw/right hindpaw (LH/RH) to remove the influence of confounding factors such as the weight and baseline level of the print area. The detection settings and gain of the camera were constant throughout the experiments for inter-animal paw comparison.

### DigiGait^™^ Analysis

DigiGait™ contains a transparent treadmill on which animals are restricted under a polymethyl methacrylate cover and forced to walk/run at a fixed velocity (usually 5 cm/s–20 cm/s) and gradient (usually 0°).

Before experiments, animals were trained to make uninterrupted runs for at least 3 step-cycles at a speed of at least 10 cm/s. Measurements were taken at 7 days or 9 days after SNI surgery or CFA injection. Each rat was placed on the treadmill repeatedly at intervals of at least 5 min to complete three uninterrupted runs (containing at least 3 step-cycles per run) for further analyses. The velocity of the treadmill was initially set at 10 cm/s and was gradually increased or decreased in 1 cm/s steps depending upon the rat’s performance, to minimize the stress induced by forced movement.

Consecutive recording (150 fps) from the ventral direction provided the following parameters for coordination and print area (projected area): (1) Coordination data (DigiGait-swing and DigiGait-duty cycle). Due to the inconvenience of DigiGait software, these coordination data were manually calculated according to the definition and formula in the CatWalk^™^ system. (2) Area data (DigiGait-projected area). In contrast to the pressured area in CatWalk^™^ acquired using the Illuminated Footprints^™^ technique, the print area in DigiGait^™^ was recognized from direct recordings of the walking/running rats from the ventral direction. Thus, the print area (projected area) in DigiGait^™^ was not merely the actual print of paw-floor contact. The DigiGait-projected area was calculated by DigiGait software in arbitrary units.

### Statistical Analysis

All of the data are presented as the mean ± SEM. Statistical analyses were performed using Prism 7.0 software (GraphPad Software, La Jolla, CA). Two groups were compared using either the unpaired or paired Student’s *t* test. Comparisons among groups receiving different treatments were made with two-way ANOVA followed by Sidak’s multiple comparisons test. Before parametric tests, the data from each group were checked with the Kolmogorov-Smirnov test and Bartlett’s test to confirm a Gaussian distribution and homogeneity of variance, respectively. Statistical significance was set at *P* < 0.05.

## Results

### Characteristics of DigiGait^™^ and CatWalk^™^ Gait Analysis Systems

**Fig. 1:**
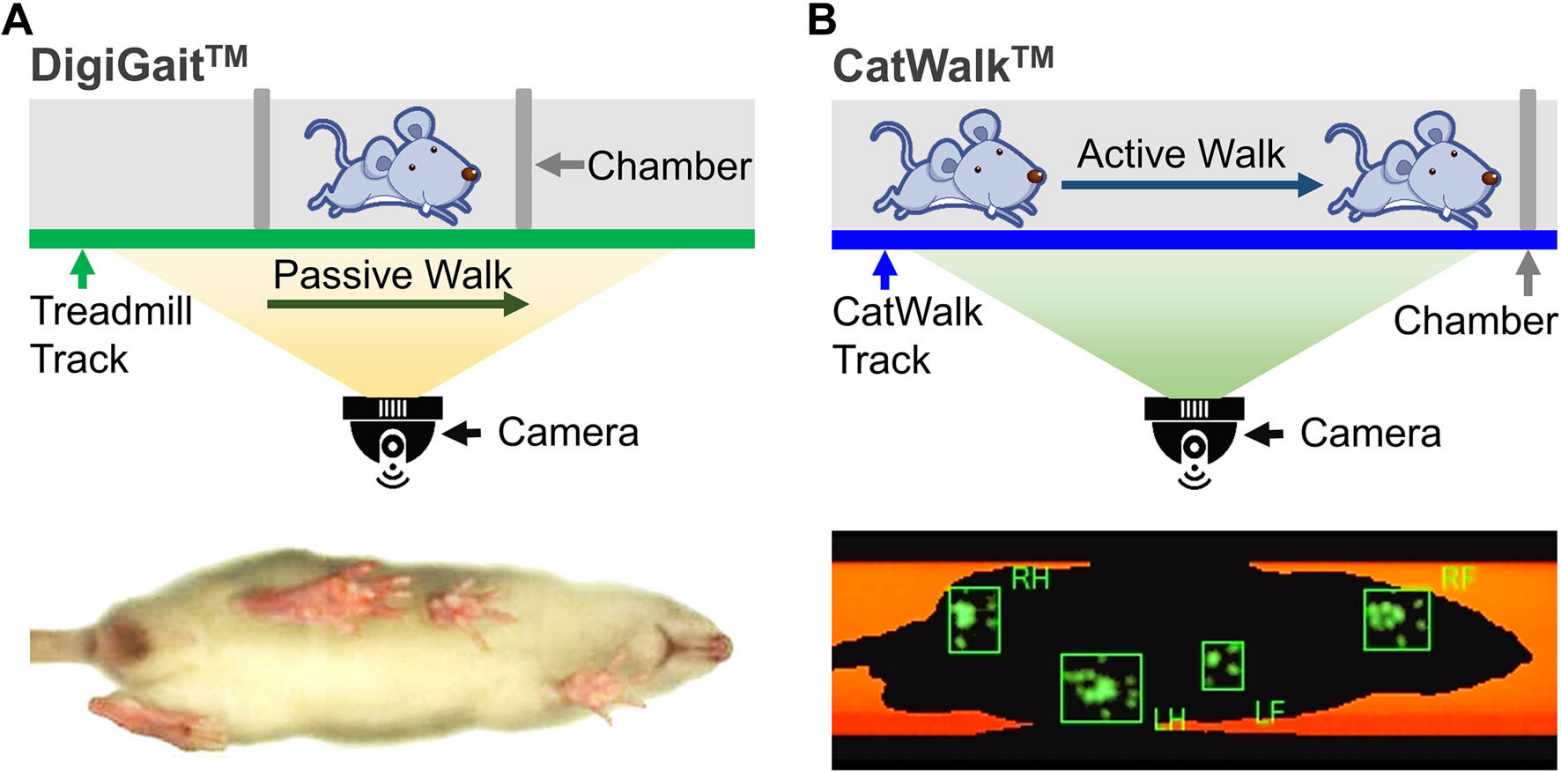
Schematic diagrams of DigiGait^™^ (**A**) and CatWalk^™^ (**B**) gait systems. The gait apparatus is outlined above and a representative image is shown below.

DigiGait^™^ contains a transparent treadmill on which animals are restricted under a polymethyl methacrylate cover and forced to walk/run at a fixed velocity (usually 5 cm/s–20 cm/s) and gradient. Consecutive recording (150 fps) from the ventral direction provides parameters concerning coordination and print area (projected area) (Fig. 1A). In the CatWalk^™^ system, the stable, horizontal walkway and relatively open space enables a rodent to actively move forward at its intrinsic speed. Of note, the Illuminated Footprints^™^ technique in CatWalk^™^ captures the light that is reflected only where the paw is physically pressing the floor, ensuring that only the actual print is measured (pressured area) and providing extra intensity data compared to DigiGait^™^ (Fig. 1B). The comparisons between DigiGait^™^ and CatWalk^™^ are summarized in Table S1.

### Efficiency of DigiGait^™^ and CatWalk^™^ in Reflecting the Severity of Neuropathic Pain in the SNI Model

**Fig. 2:**
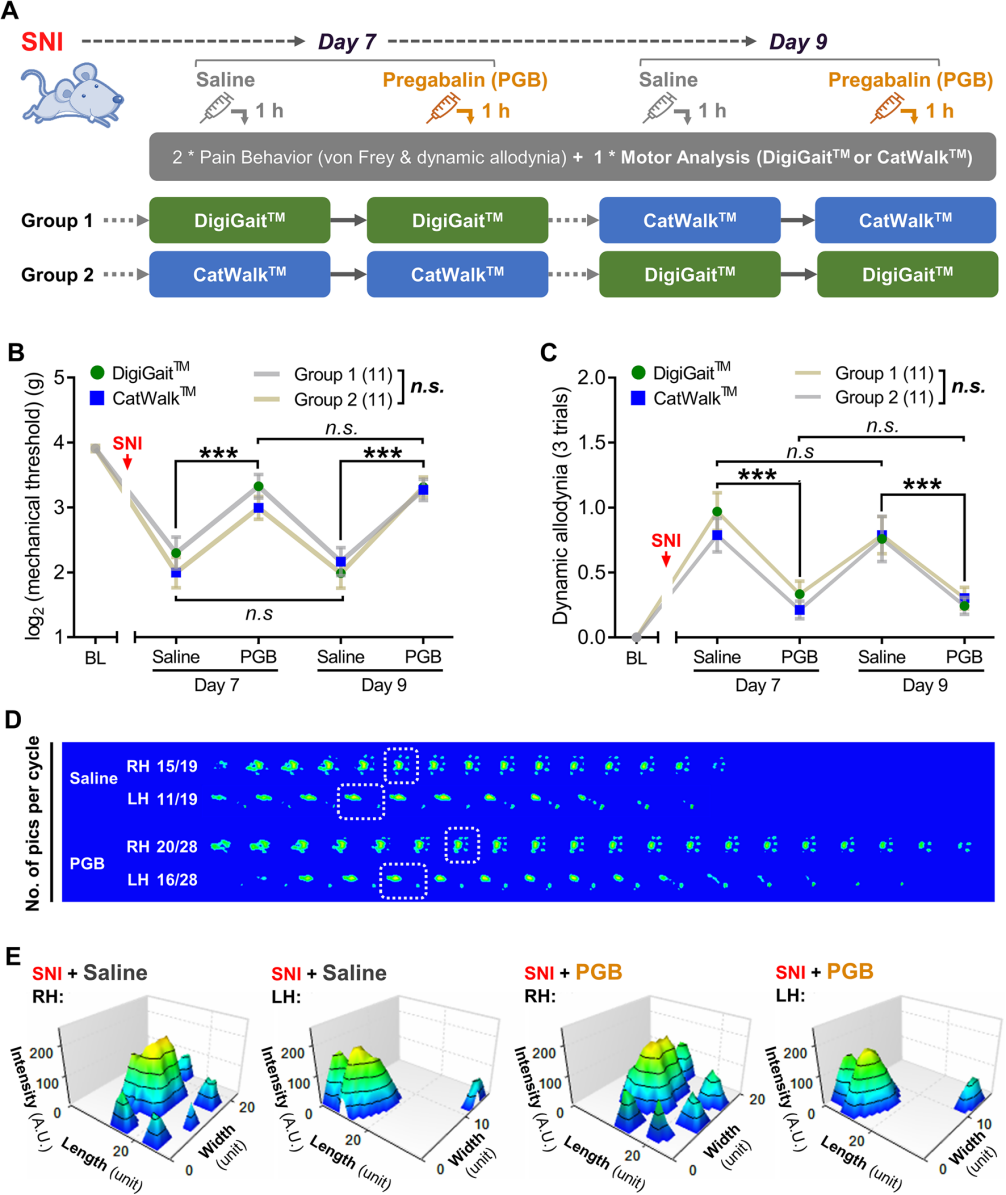
Mechanical allodynia and representative gait images in the SNI experiment. **A** Schematic diagrams of the procedures. **B** Time-course of punctate allodynia of the ipsilateral hindpaw after saline or PGB injection in SNI rats (group effect: non-significant, two-way ANOVA; post-test, ***P < 0.001, Sidak’s multiple comparisons test). **C** Time-course of dynamic allodynia of the ipsilateral hindpaw after saline or PGB injection in SNI rats (group effect: non-significant, two-way ANOVA; post-test: ***P < 0.001, Sidak’s multiple comparisons test). **D** Consecutive graphs within a duty cycle of the left hindpaw (LH) and right hindpaw (RH) after saline or PGB injection. **E** Three-dimensional graphs of LH and RH prints boxed by the dots in **D** [x-axis: length, 0.926 mm/unit; y-axis: width, 0.926 mm/unit; z-axis: intensity arbitrary units (A.U.)].

The experimental paradigm of the SNI model is shown Fig. 2A. First, the SNI operation dramatically decreased the mechanical threshold (von Frey) at 7 and 9 dpi compared to baseline, and this punctate allodynia was partially alleviated by PGB treatment in both groups (*P* < 0.001, *n* = 11). No significant difference was found between (1) the two injury groups or (2) the pre- and post-PGB status at 7 and 9 dpi, indicating similar stability of the allodynia and analgesic reactivity to PGB of these two groups in this period (Fig. 2B). Since punctate allodynia (von Frey) is mainly transmitted *via* C fibers [29, 30], the A fiber-related dynamic allodynia (brush) was tested to enrich the pain assessment in SNI rats [28, 31]. The tendency of changes in dynamic allodynia was close to that of punctate allodynia (Fig. 2C), suggesting similar functional impairments in sensory A and C fibers. In consideration of the relatively more precise procedure of von Frey assessment than brush tests, punctate allodynia was selected to represent the pain severity in the subsequent analyses.

Next, gait acquisition was conducted. Since injury was limited to the left hindlimb, the difference in paw parameters between the LH (ipsilateral side) and RH (contralateral side as control) was calculated to reflect the gait abnormalities. As in our previous studies, the LH/RH ratio was used to rule out the confounding influences of body weight and paw size [14].

For the coordination data, the time of swing, duration (swing), and the percentage in stance (duty cycle) during a single step-cycle were calculated in both DigiGait^™^ and CatWalk^™^. On the treadmill or catwalk track, rats initially contacted the floor with the palm, then transferred to the toes, and ended with the heel. Transmitted by the remaining uninjured sural nerve, mechanical hypersensitivity due to central sensitization prompted the SNI rats to avoid bearing weight on the injured left hindpaw, resulting in a longer swing duration of that limb than the uninjured right hindlimb. In representative CatWalk^™^ graphs of consecutive prints (Fig. 2D), the saline-swing (LH/RH) was 2.000, calculated as (19-11)/(19-15), and PGB decreased this ratio to 1.500, calculated as (28-16)/(28-20); the saline-duty cycle (LH/RH) was 0.733 [(11/15)/(15/19)], and PGB normalized this ratio to 0.800 [(16/28)/(20/28)].

For area data, the CatWalk^™^ print area data in each step-cycle were (1) max contact area, measured as the maximal contact area, and (2) print area, calculated as the cumulation of all footprints. In DigiGait^™^, the projected area was regarded as the largest projection of the hindpaw during stance. Representative CatWalk^™^ paw images are shown in Fig. 2D, E. In the SNI operation, incisions of tibial and common peroneal nerves led to motor dysfunction manifested as a flexed left hindpaw, contributing to (1) change in the weight-bearing area from palm to heel and (2) reduction of pressured area (Fig. 2E).

In addition, intensity data were exclusive to CatWalk^™^ owing to its Illuminated Footprints^™^ technique. The mean intensity was calculated by the average intensity of the hindpaw in a single step-cycle.

To visualize the sensory and motor data for each rat and its rank among the 22 rats, heat maps were produced based on the normalized Z score of each parameter (Fig. S1).

Given that the gait parameters might mirror the pain severity in SNI rats, colors in the same column should approximate each other. In DigiGait^™^, the colors of the mechanical threshold were similar to the coordination data, but not area data (Fig. S1A), and in CatWalk^™^, the mechanical threshold and coordination data were similar, but the intensity and area data were not (Fig. S1B).

To evaluate the discriminatory efficiency of each gait parameter, the 22 rats were classified into three groups according to their rank in the mechanical threshold (upper, median, and lower tertiles) and multiple *t* tests were then performed to determine whether the motor data of the upper and lower tertile groups differed significantly. In DigiGait^™^, neither the coordination data (*n* = 7, swing: *P* = 0.113; duty cycle: *P* = 0.067) nor the area data reached significance, and in CatWalk^™^, no gait parameter exhibited a significant difference, possibly on account of an outlier (fourth column from the right in Fig. S1).

**Fig. 3:**
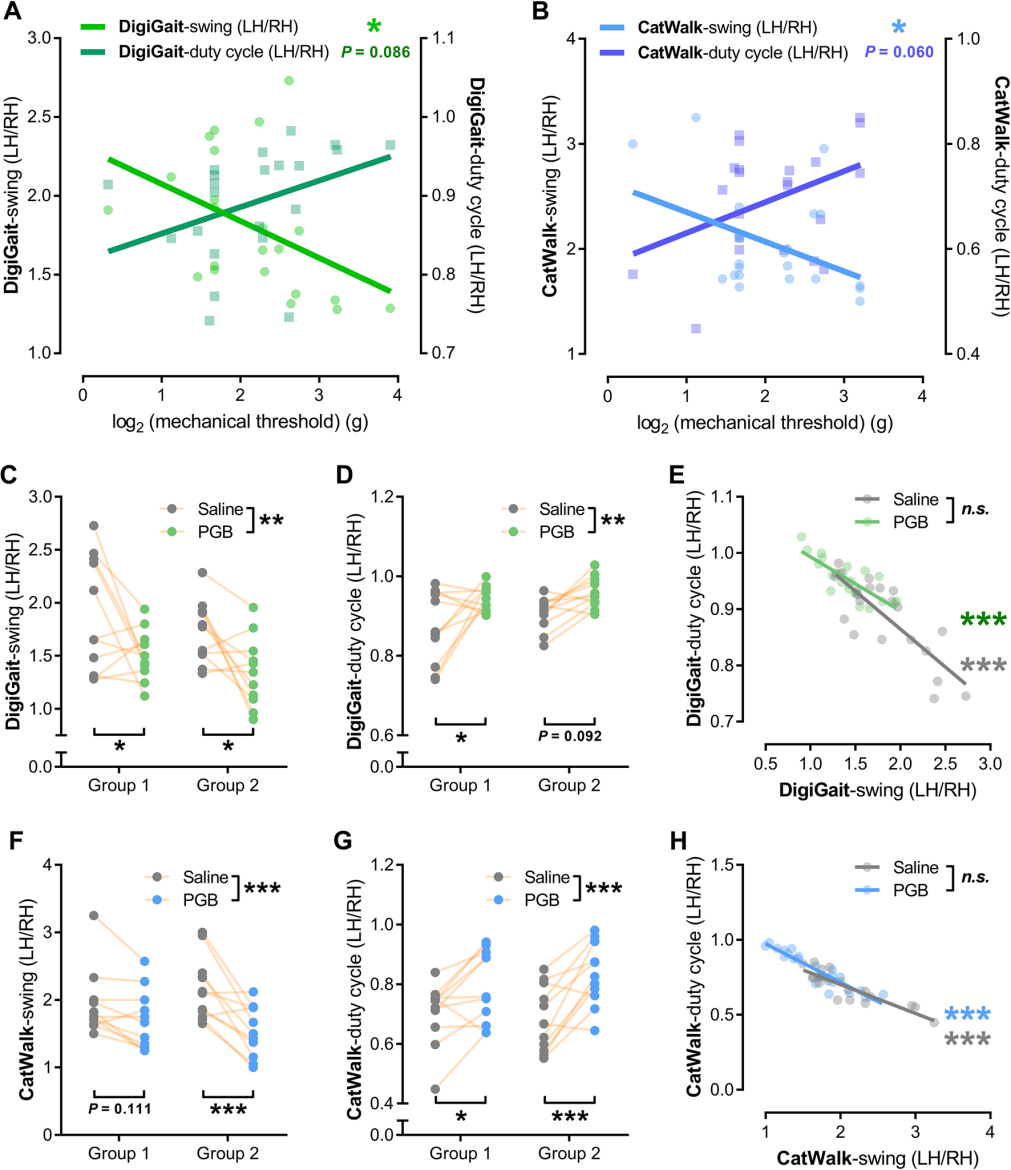
Coordination data are pain-related gait parameters in the SNI model. **A** Correlation analysis between mechanical threshold (x-axis) and DigiGait-swing (left y-axis) and DigiGait-duty cycle (right y-axis). Linear regression, *P < 0.05. **B** Correlation analysis between mechanical threshold (x-axis) and CatWalk-swing (left y-axis) and CatWalk-duty cycle (right y-axis). Linear regression, *P < 0.05. **C**, **D** DigiGait-swing (**C**) and DigiGait-duty cycle (**D**) in SNI rats after saline or PGB injection. Two-way ANOVA, treatment effect: **P < 0.01, shown in the upper right corner; post-test: Sidak’s multiple comparisons test, *P < 0.05, shown below the data points. **E** Correlation between DigiGait-swing and DigiGait-duty cycle. Linear regression, ***P < 0.001; post-test to compare the lines: non-significant. **F**, **G** CatWalk-swing (**F**) and CatWalk-duty cycle (**G**) in SNI rats after saline or PGB injection. Two-way ANOVA, treatment effect: ***P < 0.001, shown in the upper right corner; post-test: Sidak’s multiple comparisons test, *P < 0.05, ***P < 0.001, shown below the data points. **H** Correlation between CatWalk-swing and CatWalk-duty cycle. Linear regression, ***P < 0.001; post-test to compare the lines: non-significant.

**Fig. 4:**
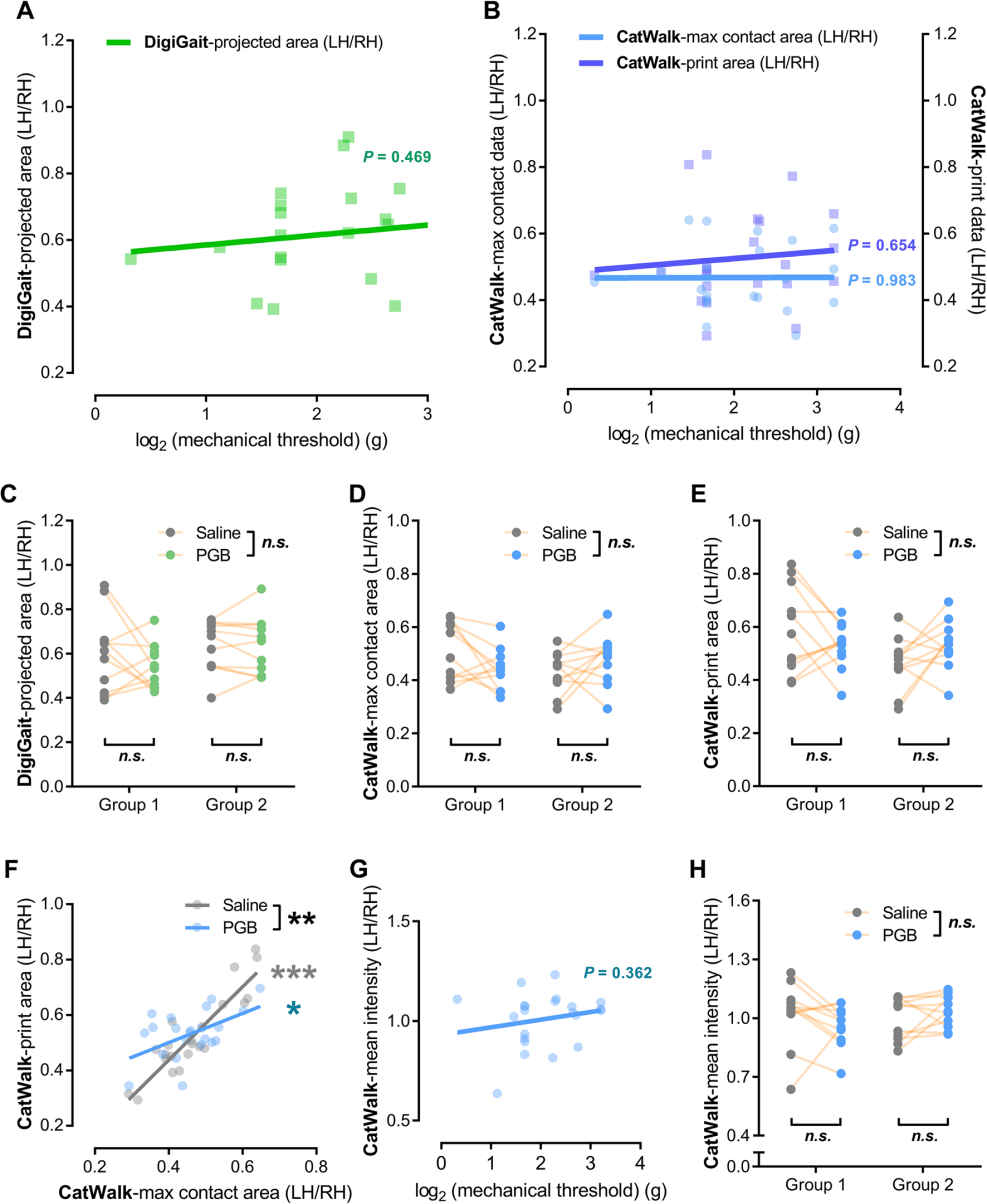
Area and intensity data failed to satisfy the criteria for pain-related gait parameters in the SNI model. **A** Correlation analysis between mechanical threshold (x-axis) and DigiGait-projected area (y-axis). Linear regression. **B** Correlation analysis between mechanical threshold (x-axis) and CatWalk-max contact area (left y-axis) and CatWalk-print area (right y-axis). Linear regression. **C**–**E** DigiGait-projected area (**C**), CatWalk-max contact area (**D**) and CatWalk-print area (**E**) in SNI rats after saline or PGB injection. Two-way ANOVA, treatment effect: non-significant, shown in the upper right corner; post-test: Sidak’s multiple comparisons test, non-significant, shown below the data points. **F** Correlation between CatWalk-max contact area and CatWalk-print area. Linear regression, ***P < 0.001; post-test to compare the lines: **P < 0.01. **G** Correlation analysis between mechanical threshold (x-axis) and CatWalk-mean intensity (y-axis). Linear regression. **H** CatWalk-mean intensity in SNI rats after saline or PGB injection. Two-way ANOVA, treatment effect: non-significant, shown in the upper right corner; post-test: Sidak’s multiple comparisons test, non-significant, shown below the data points.

Furthermore, we analyzed the correlations between mechanical threshold and each gait parameter in DigiGait^™^ and CatWalk^™^. For the coordination data in both systems, significant correlations were found for the duration of swing (*n* = 22, DigiGait^™^: *P* = 0.046; CatWalk^™^: *P* = 0.049) but not for duty cycle (*n* = 22, DigiGait^™^: *P* = 0.086; CatWalk^™^: *P* = 0.060) (Fig. 3A, B). For the area data, DigiGait^™^-projected area and CatWalk^™^-max contact area and print area displayed no correlation with punctate allodynia (Fig. 4A, B). In addition, no correlation was detected with CatWalk^™^-mean intensity (Fig. 4G). Taken together, these statistics indicated that coordination data (swing) reflected the mechanical hypersensitivity in SNI rats, but the intensity and area data did not.

The major differences between DigiGait^™^ and CatWalk^™^ were the pressured area data and extra intensity data provided in CatWalk^™^, while this advantage had no profound influence in SNI rats because these two categories of parameters were uncorrelated with mechanical threshold. Thus, it can be inferred that both the DigiGait^™^ and CatWalk^™^ gait systems might be appropriate for reflecting pain severity in the SNI model.

### Efficiency of DigiGait^™^ and CatWalk^™^ in Detecting Alleviation of Gait Abnormalities in Response to Analgesic Treatment in the SNI Model

Apart from their ability to reflect pain severity in animal models, the gait parameters are believed to be sensitive to alterations of the pain threshold in order to explore or verify the function of potential analgesics.

PGB (Lyrica®, Pfizer), FDA-approved for peripheral and central neuropathic pain, and especially associated with diabetic peripheral neuropathy, postherpetic neuralgia, and spinal cord injury in adults, was administered as an analgesic in the 22 SNI rats [32, 33]. PGB is an analogue of γ-aminobutyric acid with high affinity and selectivity for the α2δ subunit of voltage-sensitive Ca^2+^ channels (~217 amino-acids) and inhibits their activity during pain transduction [34]. Adverse effects of PGB such as balance disorder (~4%) and peripheral edema (8%) might confuse the observation of changes in gait parameters [35]. However, the balance disorder is relatively severe and cannot be counteracted by calculations, so it is necessary to determine the proper dose of PGB with moderate analgesic action and minimal adverse effects. The maximal plasma level of PGB occurs 1 h–2 h after i.p. administration [36]. In addition, the strongest analgesic action and limited side-effects occur during this period. Previous studies of PGB in a partial sciatic nerve ligation model have confirmed a slight (12.5 mg/kg) and moderate (25 mg/kg) alleviation of punctate allodynia (von Frey) and no significant reduction of rotarod staying time at 1 h post-administration of 25 mg/kg [37]. Another study in an SNI model revealed that PGB induces mild (10 mg/kg) and moderate (30 mg/kg) relief of punctate allodynia at 2 h after treatment, and 10 mg/kg–30 mg/kg had no effect on gait measures acquired using the ink-footprint strategy (toe spread, stride length, limb rotation, and print length). However, significant impairment of rotarod performance occurs after 30 mg/kg i.p. administration [36]. Taking both analgesia and the adverse effects into consideration, the dose of PGB selected was 25 mg/kg i.p., and the behavioral assessments were performed 1 h–2 h after PGB injection when its blood concentration peaked.

As described above, PGB markedly alleviated neuropathic pain in SNI rats at both 7 and 9 dpi (Fig. 2B, C). Consistent with previous correlation analyses demonstrating significant correlations between mechanical threshold and coordination data, but not intensity and area data, PGB only rescued the coordination data in both DigiGait^™^ (swing: *n* = 22, *P* = 0.002; post-test: group 1: *n* = 11, *P* = 0.044; group 2: *n* = 11, *P* = 0.047; duty cycle: *n* = 22, *P* = 0.002; post-test: group 1: *n* = 11, *P* = 0.018; group 2: *n* = 11, *P* = 0.092) (Fig. 3C, D) and CatWalk^™^ (swing: *n* = 22, *P* < 0.001; post-test: group 1: *n* = 11, *P* = 0.111; group 2: *n* = 11, *P* < 0.001; duty cycle: *n* = 22, *P* < 0.001; post-test: group 1: *n* = 11, *P* = 0.024; group 2: *n* = 11, *P* < 0.001) (Fig. 3F, G), but not the intensity (Fig. 4C–E) or area data (Fig. 4H).

The swing and duty cycle are both part of the coordination data, and they correlated with each other in linear regression analysis. To discover whether their inter-relations were altered by analgesics, the slopes of the linear regressions between the coordination data (x-axis: swing; y-axis: duty cycle) in each gait system were compared between the before-PGB and after-PGB states. In both DigiGait^™^ and CatWalk^™^, no significant difference occurred with PGB treatment. Similarly, in CatWalk^™^, this analysis was also conducted between two kinds of area data (x-axis: max contact area; y-axis: print area). As found above, the area data were uncorrelated with mechanical threshold in SNI rats and remained abnormal after PGB injection. However, to our surprise, this slope decreased substantially from 1.321 to 0.527 (*P* = 0.002) (Fig. 4F).

In view of the limited connection between area data and pain severity in SNI rats, we presumed that this significant reduction might be attributable to the side-effects of PGB, so we retrieved more original data for further analyses by hindsight. The side-effects induced by systemic administration are considered to influence both hindpaws, so we separated the LH/RH ratio into LH and RH to determine whether PGB regulated the pressure area of hindpaws differentially on the two sides. For CatWalk^™^-max contact area, moderate augmentation was found in both LH (*n* = 22, *P* < 0.001) and RH (*n* = 22, *P* < 0.001) on a similar scale (LH: from 0.913 to 1.126, 1.233-fold *versus* RH: from 2.030 to 2.496, 1.230-fold) (Fig. S2A), while relatively substantial increases were revealed in CatWalk^™^-print area in LH (*n* = 22, *P* < 0.001) and RH (*n* = 22, *P* < 0.001), and the increase was more dramatic in LH than RH (LH: from 1.367 to 2.017, 1.475-fold *vs* RH: from 2.719 to 3.831, 1.409-fold) (Fig. S2B). To determine whether these increases were limited to the pressure area data acquired in CatWalk^™^, we likewise retrieved the original data of projected area in DigiGait^™^, but no extension occurred after PGB administration (Fig. S2C). The possible reasons for this inconsistency between DigiGait^™^ and CatWalk^™^ may include the distinctive techniques for gait acquisition (projected area *vs* pressure area). After more attention was paid to the original 2D images in CatWalk^™^ (Fig. 2D), we noted a prolonged step-cycle and lengthened footprint of the RH (showing explicit contact initially by the heel) after PGB injection, suggesting that the slowed intrinsic walking/running speed (*n* = 22, *P* = 0.023) (Fig. S2D) might contribute to the longer time for hindpaw extension in both width and length and therefore increased the acquired pressured area in CatWalk^™^, while in DigiGait^™^, the treadmill speed was initially set at 10 cm/s and gradually increased or decreased in 1 cm/s steps, and no significant reduction in treadmill velocity occurred after PGB administration (Fig. S2E). These findings indicate that PGB might diminish the rat’s motivation to actively walk/run in CatWalk^™^, but not modulate the compelled extension/flexion of muscles supporting passive movement in DigiGait^™^. This inference was confirmed by the prolonged intrinsic step-cycle (representing motivation) (Fig. S2G) but unaltered stride length (representing muscle function) (Fig. S2F) induced by PGB in CatWalk^™^. These results are in harmony with the previously-reported reduced locomotor activity after PGB injection in animal models and might be attributed to its pharmacological mechanism of suppressing voltage-sensitive Ca^2+^ channels and thereby reducing the synaptic efficacy of transmitting signals in the central nervous system [36]. This downregulation of brain activity also contributes to its indications for (1) adjunctive therapy for adult patients with partial onset seizures and (2) generalized anxiety disorder in adults [32].

In summary, coordination data, but not intensity or area data, were correlated with neuropathic pain severity and sensitivity to analgesic treatment in the SNI model, demonstrating no difference between DigiGait^™^ and CatWalk^™^ in assessments of neuropathic pain in an animal. However, the higher-quality images and greater number of retrievable parameters in CatWalk^™^ might provide a slight superiority in explicitly illustrating hindpaw abnormalities and analyzing subtle alterations in hindsight when confronting unusual deviations.

### Efficiency of DigiGait^™^ and CatWalk^™^ in Reflecting the Severity of Inflammatory Pain in the CFA Model

**Fig. 5:**
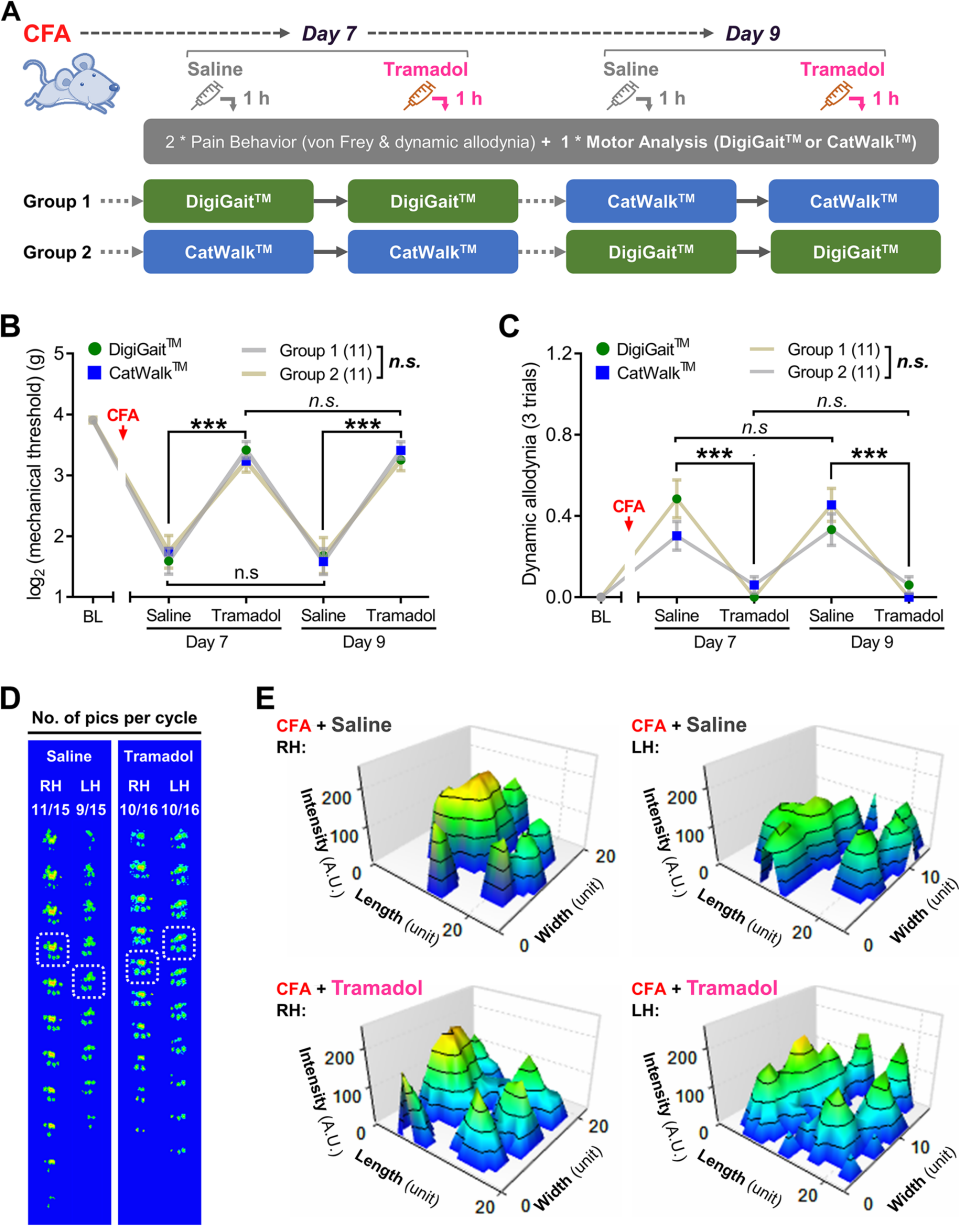
Mechanical allodynia and representative gait images in the CFA model. **A** Schematic diagrams of the procedures. **B** Time course of punctate allodynia of the ipsilateral hindpaw after saline or tramadol injection in CFA rats. Two-way ANOVA, group effect: non-significant; post-test: Sidak’s multiple comparisons test, ***P < 0.001. **C** Time course of the dynamic allodynia of the ipsilateral hindpaw after saline or tramadol injection. Two-way ANOVA, group effect: non-significant; post-test: Sidak’s multiple comparisons test, ***P < 0.001. **D** Consecutive graphs within a duty cycle of the left hindpaw (LH) and right hindpaw (RH) after saline or tramadol injection. **E** Three-dimensional graphs of LH and RH print boxed by the dotted boxes in **D**. x-axis: length (0.926 mm per unit); y-axis: width (0.926 mm per unit); z-axis: intensity, arbitrary units (A.U.).

The experimental paradigm of the CFA model is shown in Fig. 5A. Punctate and dynamic allodynia were higher at both 7 and 9 dpi than baseline and this was alleviated by tramadol (an FDA-approved analgesic for pain management). Here, in the CatWalk^™^-consecutive prints, the saline-swing (LH/RH) was 1.500, calculated as (15-9)/(15-11), and tramadol decreased this ratio to 1.000, calculated as (16-10)/(16-10); the saline-duty cycle (LH/RH) was 0.818, calculated as (9/15)/(11/15), and tramadol normalized this ratio to 1.000, calculated as (10/16)/(10/16) (Fig. 5D). In CatWalk^™^-contact print 3D images, after intraplantar injection of CFA, reduced intensity and unchanged pressured area occurred in the LH, and this reduction was rescued by tramadol (Fig. 5E).

Discriminatory efficiency was illustrated in heat maps and analyzed by multiple *t* tests between the upper and lower tertiles according to the ranking of mechanical threshold (procedures were identical to the SNI experiment described above). Coordination data, but not area data, displayed significant discriminability (*n* = 22, DigiGait^™^-swing: *P* = 0.004; DigiGait^™^-duty cycle: *P* = 0.004; CatWalk^™^-swing: *P* = 0.003; CatWalk^™^-duty cycle: *P* = 0.132) (Fig. S3A, B). Of note, inconsistent with the SNI results, CatWalk^™^-mean intensity was significantly higher in the upper tertile (*n* = 22, *P* = 0.031) (Fig. S3B), suggesting that the intensity data also reflect pain severity in the CFA model, in addition to the coordination data.

**Fig. 6:**
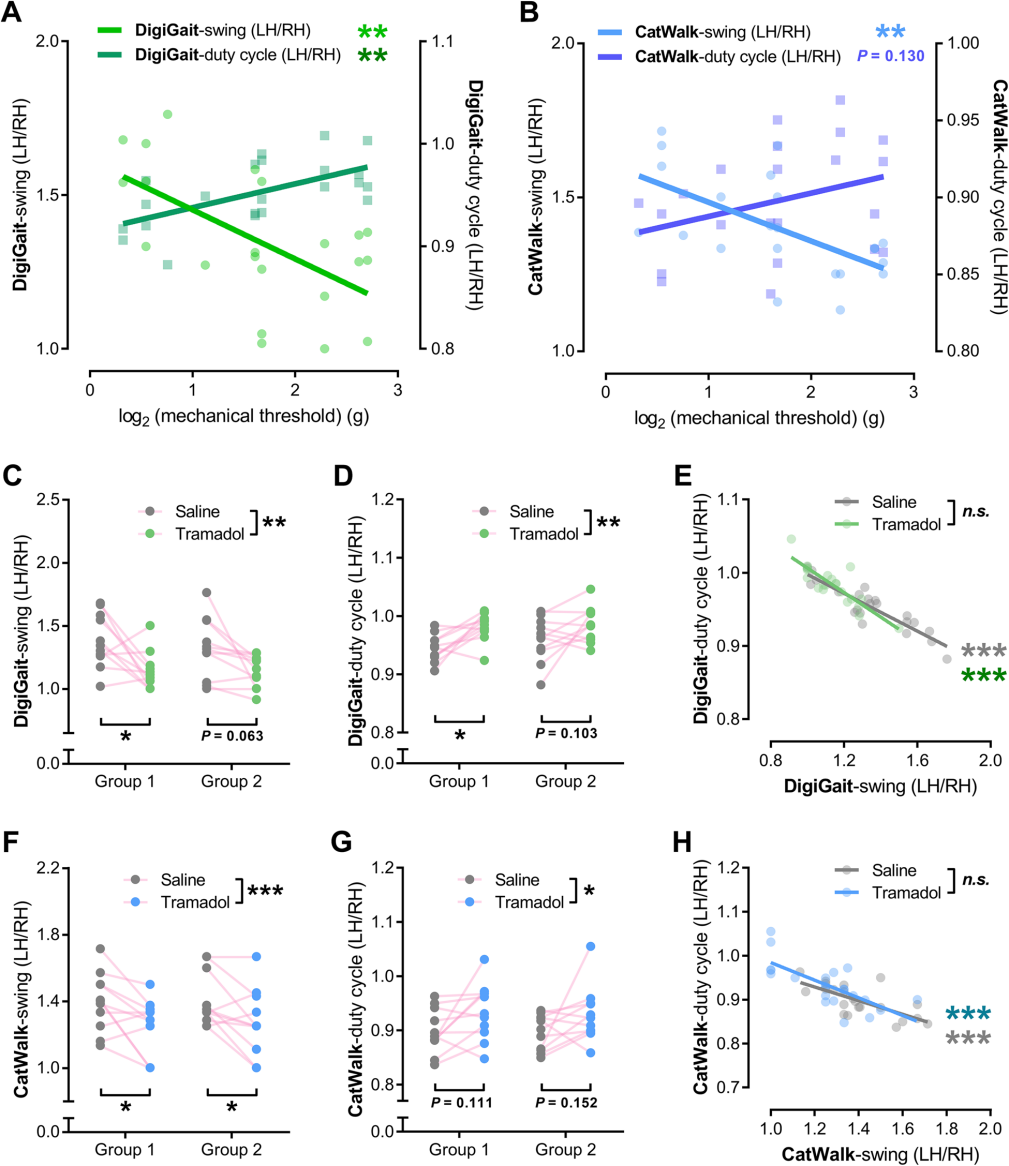
Coordination data are pain-related gait parameters in the CFA model. **A** Correlation analysis between mechanical threshold (x-axis) and DigiGait-swing (left y-axis) and DigiGait-duty cycle (right y-axis). Linear regression, **P < 0.01. **B** Correlation analysis between mechanical threshold (x-axis) and CatWalk-swing (left y-axis) and CatWalk-duty cycle (right y-axis). Linear regression, **P < 0.01. **C**, **D** DigiGait-swing (**C**) and DigiGait-duty cycle (**D**) in CFA rats after saline or tramadol injection. Two-way ANOVA, treatment effect: **P < 0.01, shown in the upper right corner; post-test: Sidak’s multiple comparisons test, *P < 0.05, shown below the data points. **E** Correlation between DigiGait-swing and DigiGait-duty cycle. Linear regression, ***P < 0.001; post-test to compare the lines: non-significant. **F**, **G** CatWalk-swing (**F**) and CatWalk-duty cycle (**G**) in CFA rats after saline or tramadol injection. Two-way ANOVA, treatment effect: ***P < 0.001, *P < 0.05, shown in the upper right corner; post-test: Sidak’s multiple comparisons test, *P < 0.05, shown below the data points. **H** Correlation between CatWalk-swing and CatWalk-duty cycle. Linear regression, ***P < 0.001, post-test to compare the lines: non-significant.

**Fig. 7:**
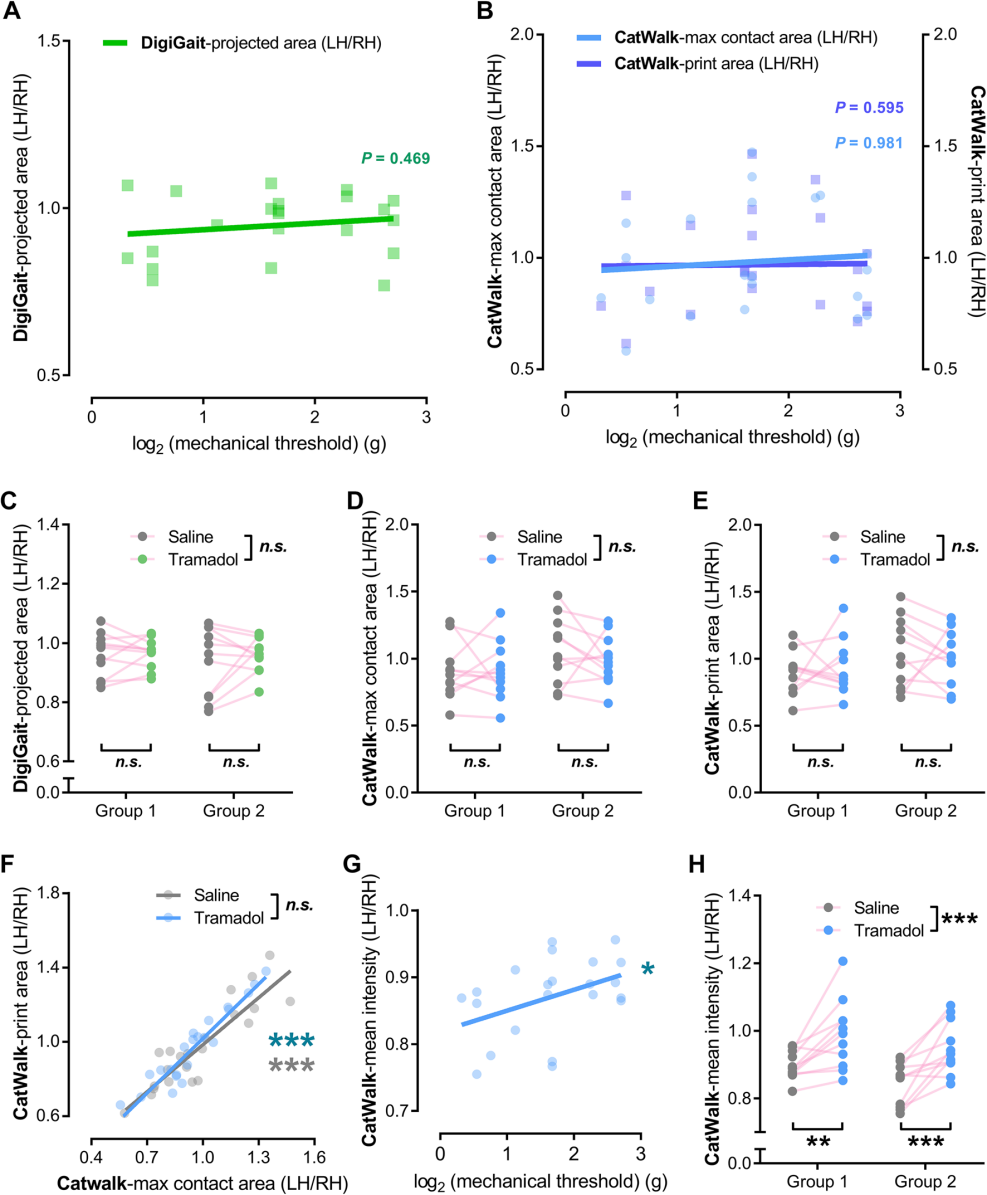
Intensity data, but not area data satisfy the criteria for pain-related gait parameters in the CFA model. **A** Correlation analysis between mechanical threshold (x-axis) and DigiGait-projected area (y-axis). Linear regression. **B** Correlation analysis between mechanical threshold (x-axis) and CatWalk-max contact area (left y-axis) and CatWalk-print area (right y-axis). Linear regression. **C**–**E** DigiGait-projected area (**C**), CatWalk-max contact area (**D**) and CatWalk-print area (**E**) in CFA rats after saline or PGB injection. Two-way ANOVA, treatment effect: non-significant, shown in the upper right corner; post-test: Sidak’s multiple comparisons test, non-significant, shown below the data points. **F** Correlation between CatWalk-max contact area and CatWalk-print area. Linear regression, ***P < 0.001; post-test to compare the lines: **P < 0.01. **G** Correlation analysis between mechanical threshold (x-axis) and CatWalk-mean intensity (y-axis). Linear regression, *P < 0.05. **H** CatWalk-mean intensity in CFA rats after saline or tramadol injection. Two-way ANOVA, treatment effect: ***P < 0.001, shown in the upper right corner; post-test: Sidak’s multiple comparisons test, **P < 0.01, ***P < 0.001, shown below the data points.

Furthermore, correlation analyses were conducted between mechanical threshold and each gait parameter in DigiGait^™^ and CatWalk^™^. For the coordination data, in both systems, strong correlations were found with duration of swing (*n* = 22, DigiGait^™^: *P* = 0.003; CatWalk^™^: *P* = 0.003) and duty cycle (*n* = 22, DigiGait^™^: *P* = 0.002; CatWalk^™^: *P* = 0.130) (Fig. 6A, B). For area data, (1) DigiGait^™^-projected area and (2) CatWalk^™^-max contact area and print area displayed no correlation with punctate allodynia (Fig. 7A, B). In addition, a significant correlation was detected with CatWalk^™^-mean intensity (*n* = 22, *P* = 0.050) (Fig. 7G). Thus, correlation statistics indicated that the coordination and intensity data, but not the area data, were correlated with the mechanical hypersensitivity in CFA rats.

According to the analyses above, CatWalk^™^ surpassed DigiGait^™^ owing to extra intensity data that correlated with mechanical threshold in the CFA model. The following experiments investigated whether these correlated gait parameters were sensitive to analgesic treatment.

### Efficiency of DigiGait^™^ and CatWalk^™^ in Detecting Alleviation of Gait Abnormalities in Response to Analgesic Treatment in the CFA Model

Tramadol (Ultram®, Janssen) was administered as the analgesic in these 22 CFA rats due to its FDA-approved indication for the management of pain in adults that is severe enough to require an opioid analgesic and for which alternative treatments are inadequate. Tramadol is a centrally-acting racemic, synthetic opioid-receptor agonist with serotonin-noradrenalin reuptake inhibitor and glutamate antagonist properties. Tramadol exists as 2 enantiomers, of which (−)-tramadol is a mild μ-receptor agonist, mild serotonin reuptake inhibitor, and moderate norepinephrine reuptake inhibitor, while (+)-tramadol strongly inhibits serotonin reuptake and activates the μ-receptor. Most importantly, the active metabolite of (+)-tramadol, O-desmethyltramadol, binds the μ-receptor with 300-fold higher affinity. Naloxone (a μ-receptor antagonist) partially blocks the analgesic action of tramadol because norepinephrine reuptake inhibition and glutamate antagonism are also related to pain relief in humans and animals [38].

Despite its adverse effects on therapeutic use including dizziness, vomiting, nausea, somnolence, and constipation, this compound still has advantages over non-steroidal anti-inflammatory drugs in the form of less renal and gastrointestinal deterioration during long-term use and over other opioid chemicals because of its lower addiction and favorable safety profile [39]. At present, a fixed combination of tramadol (37.5 mg) and acetaminophen (Ultracet®, Janssen Pharmaceuticals, Inc.) (325 mg) is popular for treating acute pain (especially postoperative pain) [40]. Considering that the slight anti-inflammatory property of acetaminophen might directly decrease the area of the swollen ipsilateral hindpaw independent of pain relief, monotherapy with tramadol was applied in the following experiments.

A previous study in rats with chronic arthritis showed that 9 mg/kg subcutaneous (s.c.) tramadol slightly reduced the mechanical allodynia, 29 mg/kg s.c. tramadol induced moderate alleviation of punctate allodynia and joint hyperalgesia, and 88 mg/kg s.c. completely rescued these sensory abnormalities to baseline level at 30 min post-administration [35]. In a sodium monoiodoacetate-induced osteoarthritis model, 100 mg/kg oral tramadol (2 h prior to tests) normalized the CatWalk^™^-max contact area and swing speed [41]. Taken together, these data led us to select the dose of 30 mg/kg tramadol, and the behavioral assessments were performed during 1 h–2 h after i.p. tramadol injection.

As described above, tramadol markedly alleviated neuropathic pain in SNI rats at both 7 and 9 dpi (Fig. 5B, C). Consistent with previous correlation analyses demonstrating significant correlations between mechanical threshold and coordination and intensity data, but not area data, tramadol only rescued the coordination data in both DigiGait^™^ (swing: *n* = 22, *P* = 0.001; post-test: group 1: *n* = 11, *P* = 0.011; group 2: *n* = 11, *P* = 0.063; duty cycle: *n* = 22, *P* = 0.002; post-test: group 1: *n* = 11, *P* = 0.012; group 2: *n* = 11, *P* = 0.103) (Fig. 6C, D) and CatWalk^™^ (swing: *n* = 22, *P* < 0.001; post-test: group 1: *n* = 11, *P* = 0.018; group 2: *n* = 11, *P* = 0.036; duty cycle: *n* = 22, *P* = 0.014; post-test: group 1: *n* = 11, *P* = 0.111; group 2: *n* = 11, *P* = 0.152) (Fig. 6F, G), and intensity data in CatWalk^™^ (*n* = 22, *P* < 0.001; post-test: group 1: *n* = 11, *P* = 0.003; group 2: *n* = 11, *P* < 0.001) (Fig. 7H), but not area data (Fig. 7C–E).

To investigate whether the inter-relations of coordination data and intensity data in each system were altered by analgesics, the slope of the linear regressions between coordination data (x-axis: swing; y-axis: duty cycle) and area data (x-axis: max contact area; y-axis: print area) were compared between the before- and after-tramadol states. Unlike the results in the SNI rats, no change in slope was found in DigiGait^™^-coordination data (Fig. 6E), CatWalk^™^-coordination data (Fig. 6H), or CatWalk^™^-area data (Fig. 7F).

In summary, coordination and intensity data, but not area data, were correlated with inflammatory pain severity and sensitive to analgesic treatment in the CFA model, indicating the superiority of CatWalk^™^ in animal studies of inflammatory pain because the intensity data can be acquired *via* the Illuminated Footprints^™^ technique in CatWalk^™^. Moreover, the CatWalk^™^-3D images of hindpaw (intensity as the z-axis) allowed us to visualize intensity together with contact area, upgrading the enrichment and intelligibility of publications.

## Discussion

### Comparison of DigiGait™ and CatWalk™ in Detecting and Illustrating Pain-Related Gait Parameters

The advantages of CatWalk^™^ include additional intensity data and corresponding 3D images compared to DigiGait^™^, due to the Illuminated Footprints^™^ technique.

In the CFA model, both coordination and intensity data, but not area data satisfied the criteria for pain-related parameters: (1) alteration in pain models, (2) correlation with nociceptive threshold, and (3) normalization by analgesics. In harmony with our results, DigiGait^™^-swing is upregulated in the inflamed hindpaw and downregulated by indomethacin and morphine [42]. Another CatWalk^™^ study revealed abnormal coordination and intensity data after intraplantar CFA injection [43]. In similar adjuvant-induced mono-arthritis models, CatWalk^™^ revealed abnormal gait data [44–54], and the intensity data were highly correlated with punctate allodynia and knee-bend score [55]. Moreover, all gait parameters were partially improved by analgesic therapies [45, 48, 50]. Taking these results together, in the CFA model, we recommend (1) coordination and intensity data, but not area data, as pain-related parameters in analyzing behavioral changes and (2) CatWalk^™^ for research involving inflammatory pain in the hindlimb, especially when impacting the distribution of weight-bearing, due to the importance of intensity data in models of plantar inflammation and osteoarthritis.

In the SNI model, coordination and area data, but not intensity data were abnormal. However, only coordination data correlated with mechanical threshold and were rectified after PGB administration, demonstrating that coordination data might serve as pain indicators in the SNI model. Consistent with our result, SNI CD-1 mice exhibited abnormal CatWalk^™^-duration of standing, paw intensity and print area in the ipsilateral hindpaw [56]. In another neuropathic pain model, the CCI model, CatWalk^™^ explored aberrant coordination, intensity and area data [13, 17, 57–59], and the alterations in coordination and intensity correlated with punctate allodynia [58]. Compared to CCI, SNI includes complete ligations and incisions of the tibial and common peroneal nerves, and therefore induces irreversible manifestations due to motor nerve injury, for instance, the flexed ipsilateral hindpaw resulting in reduced area data. Hence, in the present study, decreased area data occurred after SNI, but were uncorrelated with mechanical threshold and unaltered after analgesic therapy. The futility of intensity data in the SNI model weakens these advantages of CatWalk^™^ and the extra SFI calculated from DigiGait^™^ whole-body 2D images might provide a handy description of the degree of sciatic injury in neuropathic pain and peripheral nerve regeneration models. Taken together, we recommend that coordination data, but not intensity or area data, as pain-related parameters in analyzing behavioral changes in the SNI model, and the recommendations of the gait analysis system for models with peripheral nerve injury are based on the importance of (1) intensity data and (2) SFI *per se*. In CCI, systemic paclitaxel, epineural removal, and nerve crush models in which sciatic nerve injuries are susceptible to recovery, intensity data and SFI might be equally essential [59–62], so we recommend a combination of CatWalk^™^ and DigiGait^™^ (dynamic SFI), or CatWalk^™^ plus ink-footprint strategies (static SFI). In SNI models, neither intensity data nor SFI is a pain-related parameter, and therefore CatWalk^™^ is only slightly superior on account of its higher-quality images to explicitly illustrate hindpaw abnormalities. The comparisons between DigiGait^™^ and CatWalk^™^ in evaluating the alleviation of pain in the CFA and SNI models are summarized in Table 1.

**Table 1:** Comparisons of pain-related gait parameters of the DigiGait™ and CatWalk™ gait systems in the SNI and CFA models.

Gait parameters	Gait systems		Evaluating the alleviation of pain	
	DigiGait™	CatWalk™	SNI	CFA
Coordination data	Swing (LH/RH) Duty Cycle (LH/RH)	Swing (LH/RH) Duty Cycle (LH/RH)	Available	Available
Area data	Projected Area (LH/RH)	Max Contact Area (LH/RH) Print Area (LH/RH)	/	/
Intensity data	/	Mean Intensity (LH/RH)	/	Available
Demo image	Whole Body (2D, length * width)	Pressured Paws (3D, length * width * intensity)	/	Available
Other potential application	Nerve Injury (calculating sciatic function index as per 2D images)	Arthritis (knee) (acquiring intensity data and producing 3D demo images)		

“/” represents “not available”.

*SNI* spared nerve injury, *CFA* complete Freund’s adjuvant.

### Comparison of Fixed and Intrinsic Speed in DigiGait^™^ and CatWalk^™^ Gait Systems

Illuminated Footprints^™^ furnishes CatWalk^™^ with the ability to acquire intensity data, but meanwhile, CatWalk^™^ track lacks the ability to modulate track gradient and movement velocity. In gait analysis, velocity serves not only as a research result, but also as a factor causing considerable variability in other gait parameters [63]. Contact location and limb phasing vary with velocity, and these speed-dependent patterns are altered after spinal cord injury and gradually recover afterwards [64], indicating their potential value in reflecting the severity of nerve injury. A previous CatWalk^™^ study demonstrated that the speed-sensitive gait parameters exhibited similar alterations on both the left and right sides [63]; hence, the LH/RH ratio calculated in the present study was capable of counteracting this confounding impact of intrinsic speed, in addition to body weight and foot size [64].

Apart from calculating the LH/RH ratio, manual regulation of speed is an accessible method to adjust gait results for speed variance, for instance on a treadmill or treadwheel. However, this fixed velocity also raises two conundrums. First, forced movements do not reflect the actual intrinsic speed of the animal, but evoke stress. Since rodents are instinctive prey animals and thus unwilling to show weakness to potential predators, a treadmill or treadwheel may actually disguise the minor gait abnormalities investigated in pain research. Second, despite the same average speed shared by rodents in one test group, they might reach this average by constantly varying their speed on the treadmill with distinctive strategies, such as jumping or limping. To address these problems in DigiGait^™^, we initially set the treadmill speed at 10 cm/s and gradually increased or decreased it to minimize the stress induced by forced movement.

### Comparison of General Operations in DigiGait™ and CatWalk™ Gait Systems

Both systems require (1) preceding training and adaptation for acquiring satisfactory movement videos and (2) manually recognizing footprints to avert inaccurate analyses [65]. In addition, in CatWalk^™^, setting the appropriate imaging parameters of the Illuminated Footprints^™^ track is difficult but essential to avoid intensity signals from abdominal fur, especially in animals with severe pain. In addition, in DigiGait^™^ the contrast between paws and abdomen in rats is insufficient for automatic differentiation, unless the paws are painted with a colored liquid such as mercurochrome.

### Superiority of Gait Analysis Compared to Traditional Assessments

In the present study, we regarded gait parameters as subordinate to the mechanical threshold. However, several studies have explored the superiority of motor analysis over traditional assessments including nociceptive sensitivity and SFI. In a rat model of nerve crush plus inflammation, in which the sciatic nerve was pinched and injected with nucleus pulposus, no alteration in punctate allodynia was found, but detectable changes were revealed in coordination and area data [16]. In a CCI model, von Frey filaments failed to differentiate rats with incremental degrees of sciatic injury caused by 1, 2, 3, or 4 ligatures, while multiple coordination, intensity and area data showed significant differences between groups and notable correlations with the levels of synaptophysin and TNF-α in the dorsal root ganglia, dorsal horn, somatosensory cortex, and hippocampus [17]. In a sciatic crush model, local application of hyaluronic acid improved SFI and the angle of the ankle after 21 dpi, but detectable alleviation of mean intensity, print area and swing duration occurred much earlier, at 7 dpi [19]. To summarize, gait parameters might be correlated with malfunction of the ascending nociceptive pathway and overmatch traditional parameters in exploring subtle alterations in pain models, particularly when conventional assays encounter their ceiling or floor effects.

## Conclusion

To our knowledge, this is the first comparative study of the DigiGait^™^ and CatWalk^™^ imaging systems. We uncovered gait alterations and their responses to analgesics in representative models of neuropathic pain (SNI) and inflammatory pain (CFA). DigiGait^™^ has advantages in fixed speed and dynamic SFI (calculated from 2D whole-body images), while CatWalk^™^ excels at intrinsic velocity, intensity data (acquired by Illuminated Footprints^™^), and high-quality 3D images. In models that require both intensity data and SFI assessment (e.g., CCI, systemic paclitaxel, and crush), a CatWalk^™^ plus DigiGait^™^/ink-footprint strategy is recommended. In models that demand intensity data but not SFI (e.g., plantar inflammation and osteoarthritis), CatWalk^™^ alone is recommended. In models that warrant neither intensity data nor SFI (e.g., SNI), CatWalk^™^ is slightly superior owing to its higher-quality images to explicitly illustrate hindpaw abnormalities.

Collectively, our findings illustrate a new method to calculate the primary data from DigiGait™ into gait parameters according to the built-in formula in CatWalk™, identify pain-related gait parameters in SNI and CFA rats, and elaborate the critical distinctions between the DigiGait™ and CatWalk™ gait imaging systems. Insights into the applicability of each system may provide guidance for selecting the appropriate system for different animal models and optimization for future pain research.

## Electronic supplementary material

Below is the link to the electronic supplementary material.

Supplementary material 1 (PDF 385 kb)
