# Supplementary material for: Gait Assessment of Pain and Analgesics: Comparison of the DigiGait™ and CatWalk™ Gait Imaging Systems
Source: Neurosci Bull. 2019 Jan 18;35(3):401–18. doi: 10.1007/s12264-018-00331-y (PMC6527535; doi:10.1007/s12264-018-00331-y)
Supplement: Supplementary file 1 — Supplementary material 1 (PDF 385 kb) [file 12264_2018_331_MOESM1_ESM.pdf]

Supplementary Information

**Characteristics**	**DigiGait™**	**CatWalk™**
Track	Treadmill	Walkway
Walking Speed	Fixed	Intrinsic
Area for Rodent Activity	Closed	Relatively Open
Walking Pattern	Passive	Active
Transferring Pressure into Fluorescence Signal	Not Available	Available
Recorded Images of Paws	Projected Area	Pressured Area

Table S1 Key characteristics compared between DigiGait™ and CatWalk™ gait systems.

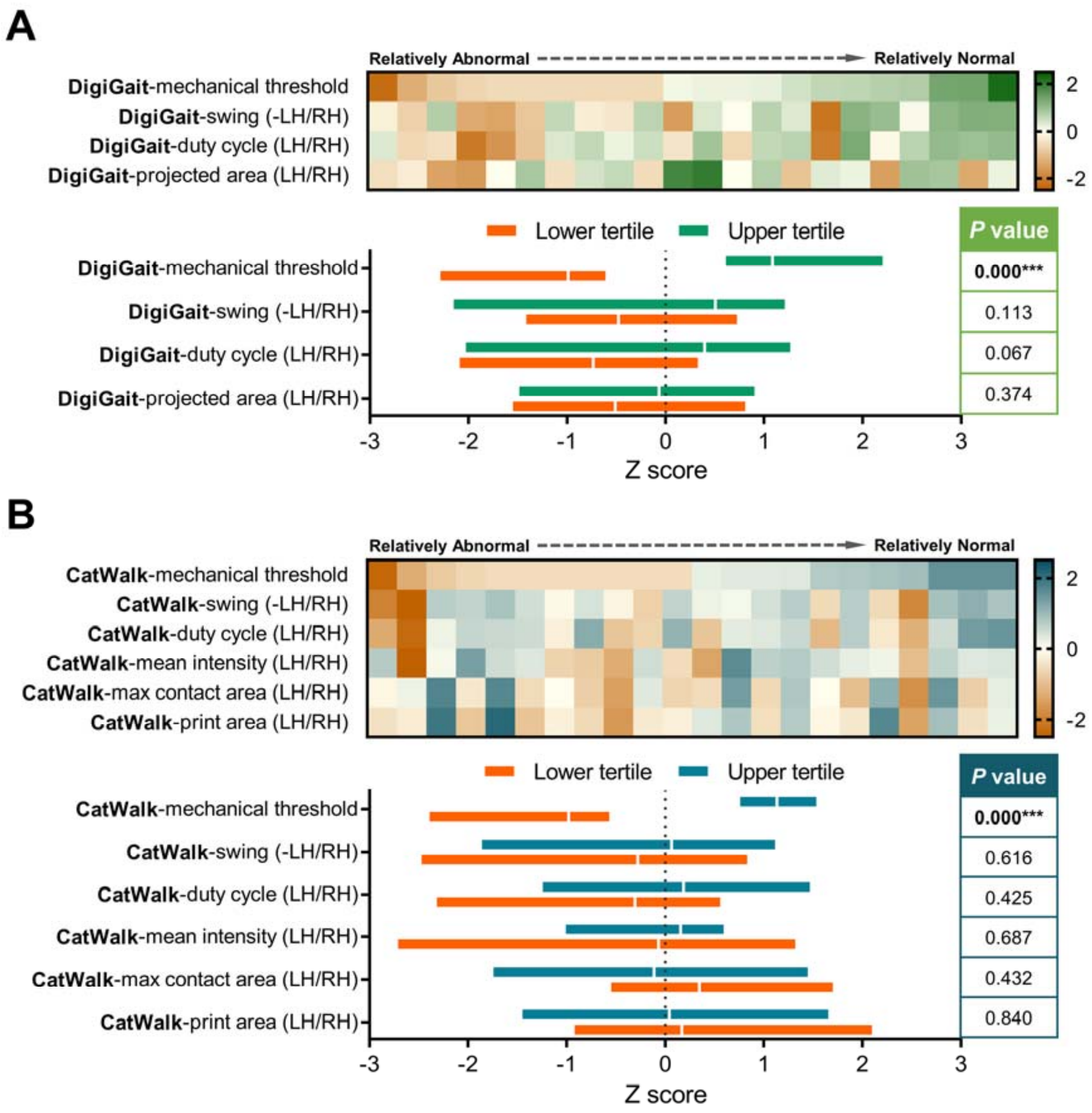

Fig. S1 Heat maps of the standardized z scores of parameters acquired by the DigiGait™ and CatWalk™ systems in SNI rats at baseline. **A** Upper panel, heat map of z scores of DigiGait™ parameters after saline injection. Lower panel, multiple *t* tests between the upper and lower tertiles of the z scores of DigiGait™ parameters defined by the mechanical threshold after saline injection. *P* values shown on right (****P* <0.001). **B** As in (**A**) for CatWalk™ parameters (****P* <0.001).

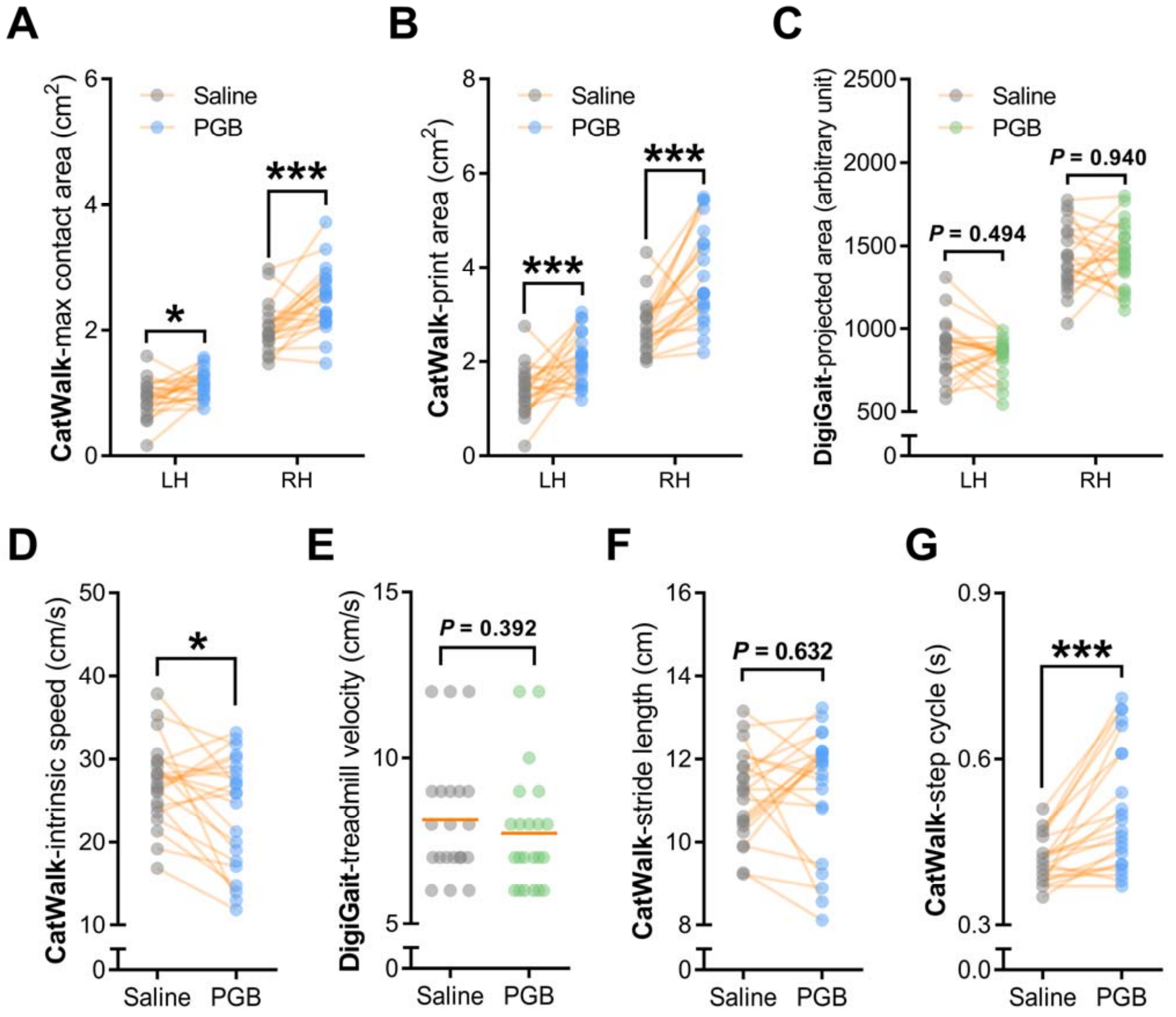

Fig. S2 Reduction of intrinsic velocity after PGB treatment in the SNI model. **A-B** CatWalk-max contact area (**A**) and CatWalk-print area (**B**) in left hindpaw (LH) and right hindpaw (RH) after saline or PGB *i.p.* injection. Paired *t* test, **P* < 0.05, ****P* < 0.001. **C** DigiGait-projected area in LH and RH after saline or PGB *i.p.* injection. Paired *t*-test. **D** CatWalk-intrinsic speed after saline or PGB *i.p.* injection. Paired *t* test, **P* < 0.05. **E** DigiGait-treadmill velocity after saline or PGB *i.p.* injection. Paired *t* test. **F** CatWalk-stride length after saline or PGB *i.p.* injection. Paired *t* test. **G** CatWalk-step cycle after saline or PGB *i.p.* injection. Paired *t* test, ****P* < 0.001.

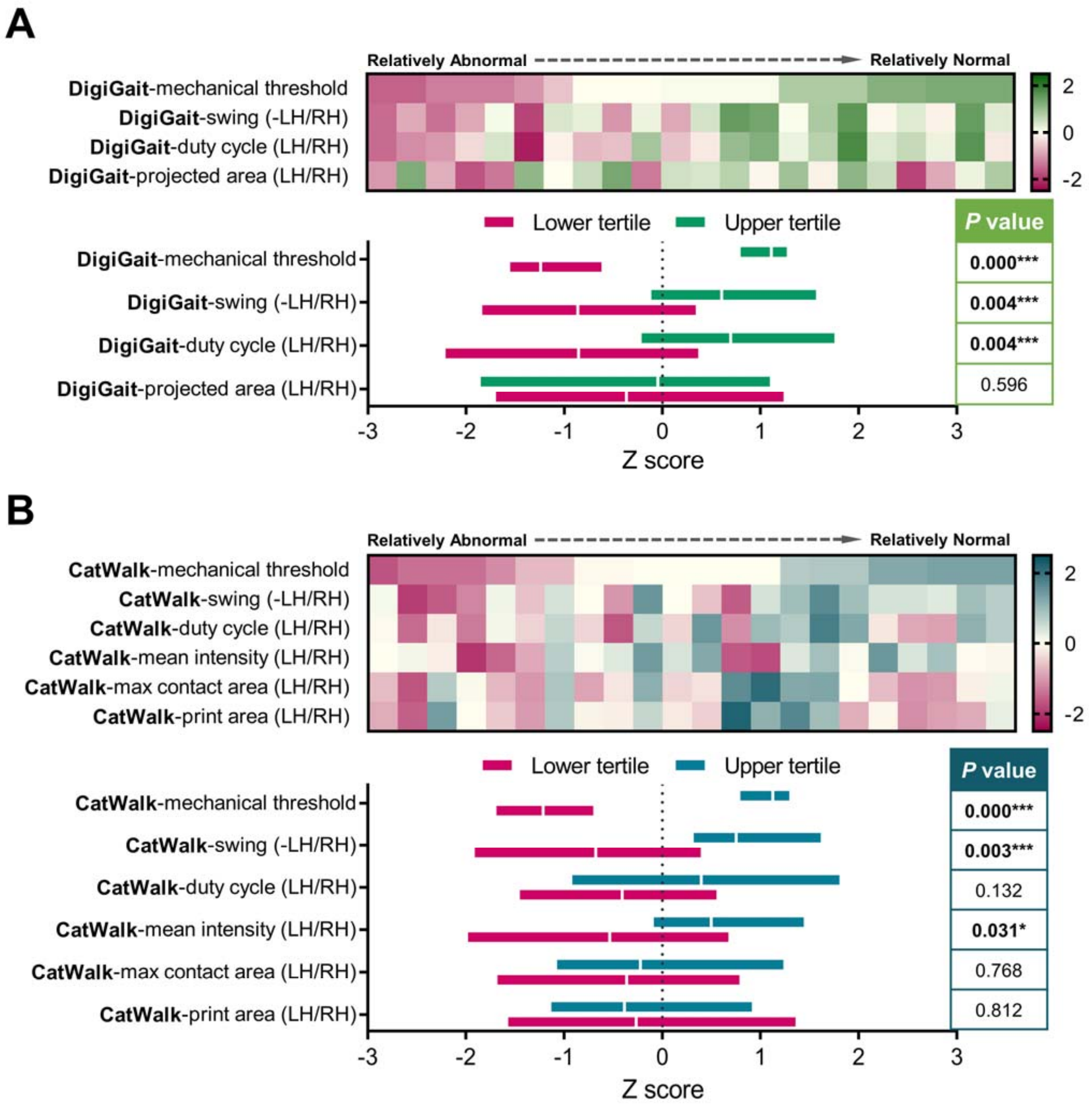

Fig. S3 Heat maps of the standardized z scores of gait parameters acquired by the DigiGait™ and CatWalk™ gait systems in CFA rats at basal status. **A** (upper) Heat map of the standardized z scores of DigiGait™ gait parameters in CFA rats after saline *i.p.* injection. (lower) Multiple *t* tests between the upper and lower tertile of the standardized z scores of DigiGait™ gait parameters in CFA rats defined by the mechanical threshold after saline *i.p.* injection. Respective *P* values shown on the right side, ***$P < 0.001$. **B** (upper) Heat map of the standardized z scores of CatWalk™ gait parameters in CFA rats after saline *i.p.* injection. (lower) Multiple *t* tests between the upper and lower tertiles of the standardized z scores of CatWalk™ gait parameters in CFA rats defined by the mechanical threshold after saline *i.p.* injection. Respective *p* values shown on the right side, *$P <$ 0.05, ***$P < 0.001$.
